# Combined effect of esaxerenone and dapagliflozin on aldosterone-mediated sodium reabsorption and potassium excretion

**DOI:** 10.3389/fphys.2025.1677518

**Published:** 2025-12-11

**Authors:** Motonobu Nakamura, Nobuhiko Satoh, Tomohito Mizuno, Mayuko Takagi, Shoko Horita, Masaomi Nangaku

**Affiliations:** 1 Division of Nephrology and Endocrinology, The University of Tokyo, Tokyo, Japan; 2 Division of Nephrology, Japan Community Healthcare Organization Tokyo Yamate Medical Center, Tokyo, Japan; 3 Teikyo University School of Medicine, Tokyo, Japan

**Keywords:** diabetic kidney disease, hyperkalemia, esaxerenone, dapagliflozin, proximal tubule

## Abstract

**Introduction:**

The efficacy of nonsteroidal mineralocorticoid receptor blockers (MRBs) in inhibiting the progression of diabetic kidney disease (DKD) is well-known. However, MRB therapy often leads to hyperkalemia and remains a major concern. Recent studies suggest that combining potassium-retaining diuretics, renin-angiotensin system inhibitors, and sodium-glucose cotransporter 2 inhibitors (SGLT2i) reduces the incidence of hyperkalemia. However, how SGLT2i, specifically affecting the proximal tubule (PT), suppresses hyperkalemia is unclear. This study aimed to elucidate the interaction between the aldosterone (Ald)/mineralocorticoid receptor (MR) signaling pathway and SGLT2i specifically in the PT, focusing on the synergistic effects on PT sodium (Na^+^) and potassium (K^+^) transport activity.

**Methods:**

We investigated the effects of Ald and SGLT2i on PT Na^+^ and K^+^ transporters. For PT Na^+^ transport function analysis, freshly isolated PTs were used to analyze luminal NHE activity and basolateral NBCe1 activity using 2′,7′-bis(carboxyethyl)-5 (6)-carboxyfluorescein acetoxymethyl ester. A DKD model was established using spontaneously diabetic Torii (SDT) fatty rats. The model rats were randomly assigned to the following groups: esaxerenone (Esx) monotherapy and Esx + dapagliflozin (Dapa) therapy. We then evaluated histological parameters, K^+^ channel expression, and various biological parameters.

**Results:**

Ald increased not only the activity of NBCe1 and NHE3 but also the expression of TWIK-1/*Kcnk1* and TASK-2/*Kcnk5*. These stimulatory effects were completely suppressed by ESX. Rats treated with Ald alone exhibited hypertension, hyperinsulinemia, and severe kidney injury, which were ameliorated by ESX; however, these rats also presented with hyperkalemia. The ESX + Dapa therapy reduced the incidence of hyperkalemia and improved kidney injury compared to ESX alone. The expression of TWIK-1 and TASK-2 increased in rats continuously treated with Ald compared with that in control rats, whereas their expression decreased to control levels in rats continuously treated with ESX alone. TWIK-1 expression did not significantly decrease in rats continuously treated with ESX and Dapa compared with that in rats treated with ESX alone.

**Discussion:**

The findings indicate that Ald stimulates Na^+^ transport via the MR in the PT and regulates the expression of K^+^ channel genes. The MRB and SGLT2i combination may mitigate MRB-induced hyperkalemia, potentially by regulating TWIK-1 expression and maintaining K^+^ homeostasis.

## 1 Introduction

Diabetic kidney disease (DKD) progression is closely associated with an increased risk of cardiovascular and renal events. Although renin-angiotensin system inhibitors (RAS inhibitors) are a cornerstone of DKD therapy, they do not completely inhibit DKD progression. One reason for this is aldosterone (Ald) breakthrough, which occurs when the RAS inhibitors trigger angiotensin II production via a pathway not mediated by angiotensin-converting enzyme (ACE), leading to elevated Ald levels and activation of mineralocorticoid receptors (MRs), ultimately resulting in organ damage (Kawarazaki and Fujita, 2016; Chrysostomou et al., 2006; Struthers and MacDonald, 2004; Naruse et al., 2002). Therefore, MR activation by Ald may be a therapeutic target for inhibiting the progression of organ damage. Spironolactone, a first-generation MR blocker (MRB), demonstrates cardioprotective and renoprotective effects and inhibits chronic kidney disease (CKD) progression effectively (Pitt et al., 1999). Eplerenone, a second-generation MRB, when administered with RAS inhibitors substantially reduces albuminuria incidence in patients with non-diabetic CKD. Recently, the addition of a third-generation MRB, esaxerenone (ESX), to existing RAS inhibitor therapy has been demonstrated to reduce microalbuminuria incidence in patients with type 2 diabetes with microalbuminuria (Ito et al., 2020).

The use of RAS inhibitors and MRBs is associated with a high risk of concurrent hyperkalemia, particularly in patients with diabetes (Palmer and Clegg, 2015; Liamis et al., 2013; Raebel et al., 2010; Jarman and Mather, 2003). Large-scale clinical trials have shown that combining ACE inhibitors with angiotensin receptor blockers (ARBs) or aliskiren fumarate, a direct renin inhibitor, in patients with diabetes fails to inhibit nephropathy development. Instead, the combined treatments were associated with increased side effects and severe hyperkalemia, leading to the premature termination of the studies (Fried et al., 2013; Parving et al., 2012). These findings highlight hyperkalemia as a critical rate-limiting factor in therapies targeting CKD progression and diabetic nephropathy.

A subanalysis of recent large-scale clinical trials on sodium–glucose cotransporter 2 inhibitors (SGLT2is), which demonstrated their efficacy in reducing the incidence of cardiovascular disease or progression of diabetic nephropathy, revealed that the incidence of hyperkalemia was lower in the SGLT2i-treated group than in the placebo group of patients with an estimated glomerular filtration rate of <60 mL/min/1.73 m^2^ (Kadowaki et al., 2019; Yavin et al., 2016).

According to classical interpretations, approximately 70% of potassium (K^+^) filtered by the glomerulus is reabsorbed in the proximal tubules (PTs) and secreted in the distal nephrons. This secretory mechanism is strongly affected by Ald, which activates the renal outer medullary potassium channel and epithelial sodium (Na^+^) channel in principal cells of the cortical-collecting ducts via MR, enhances K^+^ secretion and Na^+^ reabsorption, and induces insulin resistance. Ald may strongly contribute to the occurrence of metabolic syndrome, heart failure, and CKD via the activation of MR (Nishiyama, 2019; Joseph et al., 2018; Bochud et al., 2006).

The kidney plays a pivotal role in maintaining K^+^ homeostasis, primarily through reabsorption and secretion in the renal tubules. This process involves various K^+^ channels and transporters. Among these, the two-pore-domain K^+^ (K2 P) channels, particularly TWIK-1 (*Kcnk1*) and TASK-2 (*Kcnk5*), have important roles in regulating renal K^+^ transport (L'Hoste et al., 2007; Wang, 2004). TWIK-1 is highly expressed in the PTs and is involved in K^+^ recycling across the apical membrane, a process critical for maintaining the electrochemical gradient necessary for active transport. TASK-2 is also localized to the PTs, and its expression is known to be regulated by pH and cell volume. Thus, TASK-2 contributes to basolateral K^+^ conductance and overall tubular transport function. Ald, a key hormone in the renin-angiotensin-Ald system, is known to modulate the expression and activity of various ion channels, including those involved in regulating K^+^ level. Given the crucial roles of TWIK-1 and TASK-2 in PTs, we hypothesize that their expression is considerably affected by Ald and that SGLT2 inhibition can modulate their regulatory effect, thereby contributing to the K^+^ level-lowering effects.

The above-mentioned findings suggest that combining SGLT2is with RAS inhibitors or MRBs could reduce the risk of developing RAS inhibitor- or MRB-induced hyperkalemia. However, the underlying mechanisms remain unclear. Therefore, in this study, we focused on the PTs, the primary target of SGLT2is, to analyze the MR-mediated effects of Ald on K^+^ excretion, and Na^+^ reabsorption in the PTs to clarify the therapeutic potential of combining MRBs and SGLT2i for managing DKD.

## 2 Methods

### 2.1 Animal samples

All animal experiments were conducted in accordance with the Guidelines for Proper Conduct of Animal Experiments by the Science Council of Japan and the ARRIVE reporting guidelines and were approved by the Institutional Animal Care and Use Committee of the University of Tokyo (authorization number: P-19–88).

Male Sprague-Dawley (SD) and spontaneously diabetic Torii (SDT) fatty rats were obtained from CLEA Japan, Inc. (Tokyo, Japan). All rats were maintained in cages in-house under a 12-:12-h light/dark cycle and were fed a standard diet and provided drinking water containing 0.5% NaCl (MF; Oriental Yeast Co., Ltd., Tokyo, Japan) *ad libitum*. For *in vivo* experiments, SD rats and SDT fatty rats were provided the same diet and water until a unilateral nephrectomy was performed.

Finally, 35 and 21 rats were selected for *in vivo* and *ex vivo* experiments, respectively.

Rats were anesthetized with a mixture of three anesthetics: medetomidine (intraperitoneally, 0.75 mg/kg) (Sandoz, Tokyo, Japan), midazolam (intraperitoneally, 4 mg/kg) (Nippon Zenyaku Kogyo, Fukushima, Japan), and butorphanol (intraperitoneally, 5 mg/kg) (Meiji Seika Pharma, Tokyo, Japan), before being euthanized. After the experimental procedures, the rats were euthanized via intraperitoneal injection of a lethal dose of sodium pentobarbital (200 mg/kg).

### 2.2 PT isolation

PTs were isolated from SD rats as previously described (Nakamura et al., 2020a; Nakamura et al., 2015). Nephrons were extracted from 1.5-mm thick renal cortical tissue samples using microtweezers in ice-cold N-(2-hydroxyethyl) piperazine-N′-2-ethanesulfonic acid (HEPES)-buffered solution. The S2 segments of PTs were collected based on their morphological characteristics.

### 2.3 Measurements of NBCe1 activity in renal PTs from rats

NBCe1 activity was determined as previously described (Nakamura et al., 2020a; Nakamura et al., 2015). Briefly, the PT (S2 segment) fragment was manually microdissected from SD rat kidneys without collagenase treatment and transferred to a perfusion chamber under an inverted microscope. To avoid the effect of luminal transporters, PT fragments were collapsed using two holding pipettes. The luminally collapsed PT was incubated at 37 °C for 10 min with the acetoxymethyl ester form of a pH-sensitive fluorescent dye, 2′,7′-bis(carboxyethyl)-5 (6)-carboxyfluorescein acetoxymethyl ester (BCECF/AM; Dojindo Laboratories, Kumamoto, Japan), in Dulbecco’s modified Eagle medium (DMEM) (Sigma-Aldrich, St. Louis, MO, USA). Intracellular pH (pHi) was monitored using a photometry system with MetaFluor 7.7 software (Molecular Devices, Sunnyvale, CA, USA). The chamber was perfused with pre-warmed (38 °C) DMEM equilibrated with 5% CO_2_/95% O_2_ gas. Bath HCO_3_^−^ concentration was alternated from 25 to 12.5 mM in the presence and absence of Ald (Sigma-Aldrich) or other agents, including an SGK inhibitor, an ERK inhibitor, dapagliflozin (Dapa; Sigma-Aldrich), and ESX (Daiichi Sankyo Co., Ltd., Tokyo, Japan). NBCe1 activity was calculated using the rate of pHi decrease in response to bath HCO_3_^−^ reduction and the buffer capacity. The sample size used in this study was chosen as per previously published literature (Nakamura et al., 2020a; Nakamura et al., 2015). The following PTs data were excluded from the analysis: pHi reduction below 0.1, basal pH below 6.5.

### 2.4 Measurements of luminal Na^+^-H^+^ exchanger (NHE) activity in renal PTs from rats

Luminal NHE activity was determined as previously described (Mizuno et al., 2022; Nakamura et al., 2020a; Satoh et al., 2016; Endo et al., 2011; Mizuno et al., 2022; Nakamura et al., 2020a; Satoh et al., 2016; Endo et al., 2011). Briefly, the PT (S2 segment) fragment was freshly isolated in the same manner as for the NBCe1 activity measurement and attached to a glass coverslip with Cell-tak glue (Corning, One Riverfront Plaza, NY, USA). The PTs were placed in a perfusion chamber on an inverted microscope, and the tubule end was cut with capillary glass to expose the lumen. The lumen-exposed PT was incubated with 2′,7′-bis(carboxyethyl)-5 (6)-carboxyfluorescein acetoxymethyl ester in HEPES-buffered solution (144 mM Na^+^, 5 mM K^+^, 1.5 mM Ca^2+^, 1 mM Mg^2+^, 137 mM Cl^−^, 2 mM H_2_PO_4_^−^, 1 mM SO_4_^2-^, 5.5 mM glucose, 25 mM HEPES, adjusted to pH 7.4) for 10 min and the pHi was monitored using MetaFluor 7.7. A prewarmed (38 °C) HEPES-buffered solution was used for the bath perfusate, and 200 nM bafilomycin A1 (FUJIFILM Wako Pure Chemical, Osaka, Japan) was added to block the effect of vacuolar ATPase on PT transport. The perfusate was repeatedly switched from a HEPES-buffered solution to an isotonic Na^+^-free solution (144 mM N-methyl-D-glucamine, 5 mM K^+^, 1.5 mM Ca^2+^, 1 mM Mg^2+^, 137 mM Cl^−^, 2 mM H_2_PO_4_^−^, 1 mM SO_4_^2-^, 5.5 mM glucose, 25 mM HEPES, adjusted to pH 7.4) in the absence and presence of Ald or other agents, such as an SGK inhibitor, an ERK inhibitor, and ESX, all of which exhibited sufficient inhibitory activities without affecting the basal NHE activity in PTs. Luminal NHE activity was calculated using the rate of pHi decrease during Na^+^ removal and the buffer capacity. The sample size used in this study was chosen on the basis of previous studies (Mizuno et al., 2022; Nakamura et al., 2020a; Satoh et al., 2016; Endo et al., 2011). The following PT data were excluded from the analysis: pHi reduction below 0.1, basal pH below 6.5.

### 2.5 Small interfering (siRNA) RNA treatment in isolated rat PTs

Small interfering RNA treatment of isolated rat PTs was performed following established methods (Nakamura et al., 2020b; Nakamura et al., 2015). Freshly isolated PTs were treated with siRNAs against *Nr3c2* (RSS304008; Invitrogen, Carlsbad, CA, USA) at 40 nM or scrambled negative control (sc-37007; Santa Cruz Biotechnology) using Lipofectamine 2000 and Opti-MEM I Reduced Serum Medium (both from Invitrogen). The PTs were incubated in DMEM supplemented with 10% fetal bovine serum at 37 °C overnight and used for the measurement of NBCe1 and luminal NHE activities and quantitative polymerase chain reaction (PCR). The sample size used in this study was selected based on previous reports (Nakamura et al., 2020a; Nakamura et al., 2015).

### 2.6 RNA extraction and quantitative PCR analysis

Total RNA was extracted from tissues and cells using Isogen II (Nippon Gene, Tokyo, Japan) per the manufacturer’s instructions and first-strand cDNA was synthesized using a cDNA Synthesis Kit (Takara, Tokyo, Japan) as previously reported (Mizuno et al., 2022; Nakamura et al., 2020b; Nakamura et al., 2015). mRNA expression levels were estimated using quantitative PCR (Prism 7000; Applied Biosystems, Foster City, CA, USA) with TaqMan Gene Expression Master Mix (Applied Biosystems) and TaqMan Gene Expression Assay kits (Rn00565562_m1 for rat Nr3c2, Rn00582881_m1 for rat Kcnma1, Rn00583376_m1 for rat *Kcnq1*, Rn01641410_m1 for rat *Kcnk1*, Rn01755927_m1 for rat *Kcnk5*, Rn02377069_s1 for rat *Kcne3*, and Rn00667869_m1 for rat β-actin; Thermo Fisher Scientific, Waltham, MA, USA). mRNA levels were normalized to β-actin expression levels. Arbitrary units were calculated as the expression level of mRNA corrected for β-actin in the glomeruli as 1. Although mRNA expression levels may reflect protein synthesis capacity, quantitative PCR analysis does not directly assess the functional activity of the K^+^-channels. Therefore, the K^+^ channel activity cannot be confirmed through this analysis.

### 2.7 Rat experiment *in vivo*

SDT fatty rats were subjected to unilateral nephrectomy at 8 weeks and were categorized into the following groups: rats treated with a continuous pump of 0.75 μg/h Ald (dosage was based on a previous report (Nishiyama et al., 2004), n = 9); rats treated with a continuous pump of Ald at 0.75 μg/h and ESX at 3 mg/kg via gavage daily (n = 7); and rats treated with a continuous pump of Ald at 0.75 μg/h and Dapa at 10 mg/kg via gavage daily (n = 8). Male SD rats were used as controls (n = 11). The sample size of the *in vivo* experiments in this study was calculated with reference to previous reports (Nakamura et al., 2020a). Blood pressure and body weight (BW) were measured weekly. All the rats were fasted for 12 h before euthanasia. Urine output was measured over a 4-h period immediately preceding euthanasia. Ald was administered via subcutaneously implanted ALZET osmotic pumps (DURECT Corporation, Cupertino, CA, USA) for the final 30 days of the study. Forty-four rats (13 and 31 SD and SDT fatty rats, respectively) were assigned randomly. Random numbers were generated using the standard = RAND () function in Microsoft Excel. Two rats that died within 3 days after nephrectomy were excluded from the analysis. Four and three rats that were oversedated by anesthesia and those that hemorrhaged, respectively, were excluded from the study. Finally, a total of 35 rats were included in the *in vivo* experiments. Detailed materials and methods are provided in Supplementary Methods.

### 2.8 Statistical analysis

Data are presented as means ± standard error of the mean (SEM) (raw data, Table 1). Data in Table 2 and the relevant figures are presented as least squares means (LS means) ± SEM, derived from ANCOVA. Data were analyzed with JMP Pro 17 (SAS Institute, Cary, NC, USA) using the Wilcoxon signed-rank or Kruskal–Wallis test, followed by the Steel or Steel–Dwass test. However, owing to the observed difference in body weight (BW) between the control and model groups (SD vs. SDT fatty rats), the effects of treatment on all relevant *in vivo* parameters were analyzed using ANCOVA. Experimental endpoint BW was included as a covariate.

**TABLE 1:** Effects of esaxerenone and dapagliflozin treatment for 4 weeks with a high-salt diet and continuous aldosterone administration on biological parameters in rats.

Biological parameter	SD rats	SDT fatty rats	SDT fatty rats	SDT fatty rats
Ald	–	+	+	+
ESX	–	–	+	+
Dapa	–	–	–	+
N	11	9	7	8
BW (g)	313.99 ± 6.28	452.53 ± 8.17^**^	469.97 ± 4.72^**^	420.26 ± 21.21^**†^
sBP (mmHg)	119.09 ± 1.37	186.33 ± 3.28^**^	118.57 ± 2.64^##^	115.25 ± 2.49^##^
dBP (mmHg)	73.54 ± 1.57	103.11 ± 2.79^**^	69.71 ± 1.49^##^	70.65 ± 2.12^##^
BUN (mg/dL)	21.5 ± 1.09	28.69 ± 2.24^**^	38.43 ± 2.77^**#^	30.58 ± 0.52^**^
Cre (mg/dL)	0.35 ± 0.02	0.59 ± 0.08^**^	0.31 ± 0.02^*##^	0.3 ± 0.07^##^
K (mmol/L)	4.95 ± 0.14	4.73 ± 0.16	6.20 ± 0.17^**##^	4.71 ± 0.18^‡^
HCO_3_^−^ (mmol/L)	30.7 ± 0.68	17.91 ± 1.03^**^	26.13 ± 0.69^**##^	25.99 ± 0.63^**##^
Fasting BS (mg/dL)	98.73 ± 2.49	236.56 ± 31.79^**^	218.43 ± 9.43^**^	95.0 ± 6.07^##‡^
Fasting serum insulin (µg/mL)	1.38 ± 0.059	13.51 ± 1.07^**^	3.59 ± 0.80^**##^	1.40 ± 0.095^##‡^
Heart weight (g/gBW)	3.85 ± 0.095	6.31 ± 0.49^**^	2.7 ± 0.10^**##^	2.85 ± 0.19^**##^
Kidney weight (g/gBW)	7.65 ± 0.31	12.27 ± 0.92^**^	5.24 ± 0.27^**##^	5.89 ± 0.30^**##^
FENa (%)	2.22 ± 0.40	11.97 ± 1.51^**^	2.6 ± 0.36^**##^	5.86 ± 0.93^**#‡^
Urine Alb/gCre (mg/gCre)	30.4 ± 6.88	3607.4 ± 816.70^**^	115.1 ± 14.13^**##^	75.87 ± 10.58^**##‡^
CCr (ml/min/100 g BW)	0.74 ± 0.075	0.059 ± 0.017^**^	0.17 ± 0.04^**##^	0.24 ± 0.018^**##^
Glomerular sclerosis index	1.09 ± 0.01	3.39 ± 0.04^**^	1.91 ± 0.04^**##^	1.26 ± 0.03^**##‡^
Tubular injury score	0.28 ± 0.04	1.72 ± 0.04^**^	0.90 ± 0.30^**##^	0.71 ± 0.04^**##‡^

Data are presented as mean ± standard error.

*p < 0.05 versus SD, rats subjected to unilateral nephrectomy (UNx), **p < 0.01 versus SD, rats subjected to UNx.

^#^p < 0.05 versus SDT, fatty rats subjected to UNx, and continuous aldosterone (Ald) treatment.

^##^p < 0.01 versus SDT, fatty rats subjected to UNx, and continuous Ald treatment.

^†^p < 0.05 versus SDT, fatty rats subjected to UNx, and continuous Ald treatment plus daily administration of esaxerenone (ESX).

^‡^p < 0.01 versus SDT, fatty rats subjected to UNx, and continuous Ald treatment plus daily administration of ESX.

Dapa, dapagliflozin; ACR, albumin creatinine ratio; BUN, blood urea nitrogen; BW, body weight; Cre, creatinine; sBP, systolic blood pressure; dBP, diastolic blood pressure; UV, urinary volume; BS, blood sugar.

**TABLE 2:** Adjusted biological parameters (least squares means) after controlling for body weight using ANCOVA.

Biological parameter	SD rats	SDT fatty rats	SDT fatty rats	SDT fatty rats
Ald	–	+	+	+
ESX	–	–	+	+
Dapa	–	–	–	+
N	11	9	7	8
sBP (mmHg)	126.42 ± 3.90	182.52 ± 2.85^**^	113.35 ± 3.50^##^	114.03 ± 2.48^##^
dBP (mmHg)	74.73 ± 3.57	102.50 ± 2.61^**^	68.87 ± 3.20^##^	70.43 ± 2.27^##^
BUN (mg/dL)	21.51 ± 3.00	28.69 ± 2.20	38.42 ± 2.69^*#^	30.57 ± 1.91
Cre (mg/dL)	0.29 ± 0.04	0.63 ± 0.03^**^	0.36 ± 0.03^##^	0.31 ± 0.02^##^
K (mmol/L)	4.39 ± 0.25	5.02 ± 0.18	6.59 ± 0.22^**^	4.81 ± 0.16^‡^
HCO_3_^−^ (mmol/L)	30.23 ± 1.35	18.16 ± 1.00^**^	26.47 ± 1.21^##^	26.07 ± 0.86^##^
Fasting BS (mg/dL)	79.50 ± 29.06	246.57 ± 21.28^**^	232.12 ± 26.05^*^	98.20 ± 18.45^##‡^
Fasting serum insulin (µg/mL)	1.63 ± 1.10	13.38 ± 0.80^**^	3.41 ± 0.98^##^	1.36 ± 0.70^##^
Heart weight (g/gBW)	N/A	N/A	N/A	N/A
Kidney weight (g/gBW)	5.97 ± 0.86	13.16 ± 0.63^**^	6.44 ± 0.77^##^	6.17 ± 0.55^##^
FENa (%)	4.72 ± 1.51	10.67 ± 1.11	0.84 ± 1.36^##^	5.44 ± 0.96^##†^
Urine Alb/gCre (mg/gCre)	129.088 ± 722.53	3555.94 ± 528.99^**^	44.74 ± 647.69^##^	59.42 ± 458.72^##^
CCr (mL/min/100 g BW)	0.94 ± 0.23	0.091 ± 0.12^*^	0.40 ± 0.35	0.25 ± 0.06^**^
Glomerular sclerosis index	1.15 ± 0.12	3.39 ± 0.07^**^	1.72 ± 0.19^##^	1.26 ± 0.03^##^
Tubular injury score	0.28 ± 0.16	1.70 ± 0.09^**^	0.86 ± 0.24^##^	0.72 ± 0.04^##^

Data are presented as least squares means (LS, means) ± standard error (SE) from ANCOVA.

*p < 0.05 versus SD, rats subjected to unilateral nephrectomy (UNx), **p < 0.01 versus SD, rats subjected to UNx.

^#^p < 0.05 versus SDT, fatty rats subjected to UNx, and continuous aldosterone (Ald) treatment.

^##^p < 0.01 versus SDT, fatty rats subjected to UNx, and continuous Ald treatment.

^†^p < 0.05 versus SDT, fatty rats subjected to UNx, and continuous Ald treatment plus daily administration of esaxerenone (ESX).

^‡^p < 0.01 versus SDT, fatty rats subjected to UNx, and continuous Ald treatment plus daily administration of ESX.

Dapa, dapagliflozin; ACR, albumin creatinine ratio; BUN, blood urea nitrogen; BW, body weight; Cre, creatinine; sBP, systolic blood pressure; dBP, diastolic blood pressure; UV, urinary volume; BS, blood sugar; N/A, not applicable.

Prior to ANCOVA, the assumption of homogeneity of regression slopes was tested by including the Group × BW interaction term in the full model. For parameters where the interaction term was not statistically significant (P > 0.05), the final ANCOVA model was simplified to include only the main effects of Group and BW, reflecting a common slope model. For the post hoc analysis following the detection of a significant Group main effect (P < 0.05), Tukey’s honestly significant difference test was applied to the LS means. For the histological scores, the unit of analysis was not the animal itself but the individual, non-overlapping microscopic fields or glomerulus/tubules randomly sampled from each animal (e.g., N = 1741 and N = 342 data points, respectively). This approach was used to maximize the statistical power for the comparison of tissue-level changes, resulting in the larger degrees of freedom shown in Supplementary Table S1.

Statistical significance was set at p < 0.05.

## 3 Results

### 3.1 Effect of ald on PT Na^+^ transport activity is mediated by the MR signaling pathway

**FIGURE 1:**
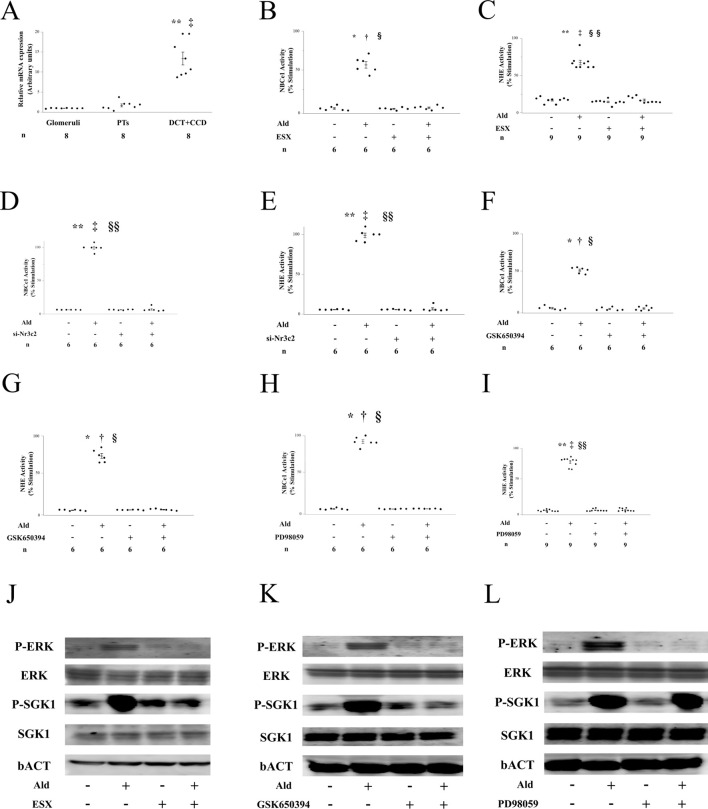
Effect of Ald on Na transport activity in proximal tubules (PTs) is mediated by the MR signaling pathway **(A)** NR3C2 expression in the kidney and mRNA expression ratios in the glomeruli, proximal tubules (PTs), and other compartments isolated manually from fresh renal cortices. DT + CCD, distal tubule and cortical collecting duct. **p < 0.01 versus glomeruli; ^‡^p < 0.01 versus PT, **(B)** Effect of 3 µM ESX on 1 nM Aldosterone (Ald)-stimulated Na-HCO_3_ cotransporter (NBCe1) activity in isolated rat PTs (n = 6), **(C)** Effect of 3 µM esaxerenone (ESX) on 1 nM Ald-stimulated Na^+^-H^+^ exchange transporter (NHE) activity in isolated rat PTs (n = 9). **(D)** Effects of small interfering RNA (siRNA) against Nr3c2 on 1 nM Ald-stimulated NBCe1 activity in isolated rat PTs (n = 6). **(E)** Effects of siRNA against Nr3c2 on 1 nM Ald-stimulated NHE activity in isolated rat PTs (n = 6), **(E)** Effect of 10 µM GSK650394 on 1 nM Ald-stimulated NBCe1 activity in isolated rat PTs (n = 6), **(G)** Effect of 10 µM GSK650394 on 1 nM Ald-stimulated NHE activity in isolated rat PTs (n = 6), **(H)** Effect of 10 µM PD98059 on 1 nM Ald-stimulated NBCe1 activity in isolated rat PTs (n = 6), **(I)** Effect of 10 µM PD98059 on 1 nM Ald-stimulated NHE activity in isolated rat PTs (n = 6), **(J)** Ald-induced SGK1 and ERK phosphorylation and the effect of ESX on their phosphorylation in the rat renal cortex, **(K)** Ald-induced SGK1 and ERK phosphorylation and the effect of GSK650394 on their phosphorylation in the rat renal cortex, **(L)** Ald-induced SGK1 and ERK phosphorylation and the effect of PD98059 on their phosphorylation in the rat renal cortex. **(B,C,F–I)** *p < 0.05 versus untreated PTs; **p < 0.01 versus untreated PTs; ^†^ p < 0.05 versus ESX-treated PTs; ^‡^ p < 0.01 versus ESX-treated PTs; § p < 0.05 versus Ald and ESX-treated PTs; §§ p < 0.01 versus Ald and ESX-treated PTs. **(D,E)** **p < 0.01 versus control PTs treated with scrambled siRNA; ^‡^ p < 0.01 versus si-Nr3c2-treated PTs; §§ p < 0.01 versus Ald and si-Nr3c2-treated PTs.

First, we examined MR expression in PTs. The MR gene *NR3C2* was expressed at similar levels in the PTs and glomeruli (Figure 1A). At physiological concentrations, Ald did not affect the pHi of PTs (Supplementary Figure S1A), whereas it increased the activity of NBCe1 and luminal NHE in PTs in a concentration-dependent manner. ESX completely inhibited the stimulatory effect of Ald on NBCe1 and NHE activities without affecting the basal activity or PT pHi (Figures 1B,C). The silencing of MR expression with siRNA in PTs completely suppressed the Ald-induced stimulation of NBCe1 and NHE activities without affecting basal activity or pHi (Figure 1D,E; Supplementary Figure S1A). Next, we examined the downstream signals of Ald. The SGK inhibitor GSK650394 and ERK inhibitor PD98059 completely suppressed the stimulatory effects of Ald on PT Na^+^ transport (Figures 1F–I). At the protein expression level, Ald phosphorylated SGK1 and ERK. While ERK phosphorylation was inhibited by ESX, GSK650394, and PD98059, SGK1 phosphorylation was inhibited by ESX or GSK650394 but not by PD98059 (Figures 1J–L; Supplementary Figures S2A-F). Ald stimulates Na^+^ transport in PTs, which is abolished by ESX and siRNA silencing, and by inhibitors of SGK1 and ERK.

### 3.2 Stimulatory effects of ald via MR on PT Na^+^ transport is regulated by SGLT2i

**FIGURE 2:**
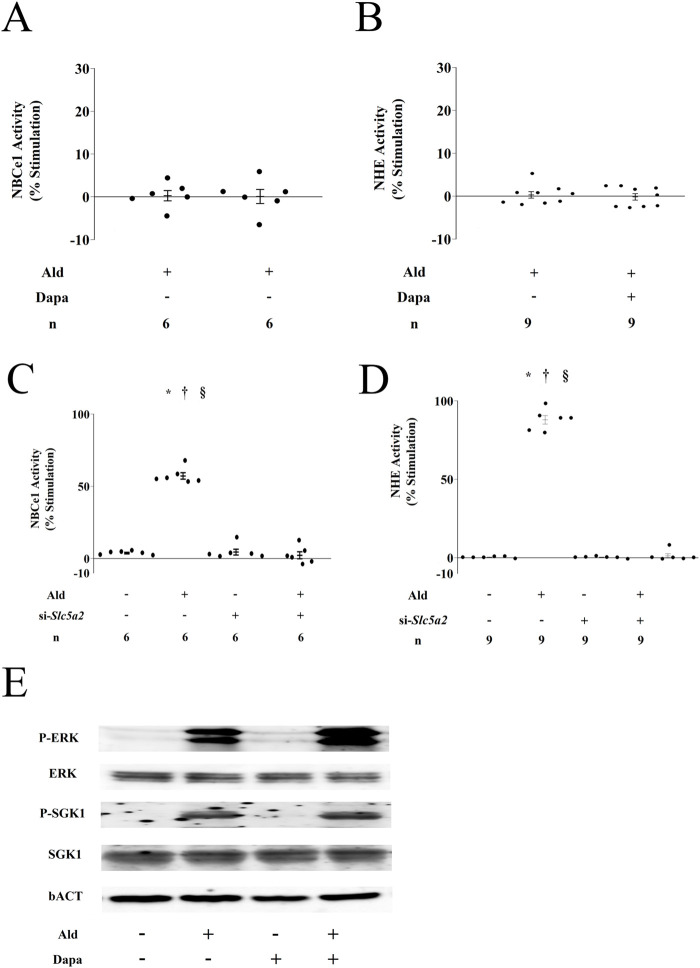
Effect of dapagliflozin *ex vivo* **(A)** Effect of 30 µM dapagliflozin (Dapa) on 1 nM aldosterone (Ald)-stimulated Na-HC_O3_ cotransporter (NBCe1) activity in isolated rat proximal tubules (PTs) (n = 6) **(B)** Effect of 30 µM Dapa on 1 nM Ald-stimulated Na^+^-H^+^ exchange transporter (NHE) activity in isolated rat PTs (n = 9) **(C)** Effects of small interfering RNA (siRNA) against Slc5a2 on 1 nM Ald-stimulated NBCe1 activity in isolated rat PTs (n = 6) **(D)** Effects of siRNA against Slc5a22 on 1 nM Ald-stimulated NHE activity in isolated rat PTs (n = 9) **(E)** Effect of Dapa on Ald-induced ERK or SGK1 phosphorylation in the rat renal cortex **(C,D)** *p < 0.05 versus control PTs treated with scrambled siRNA, ^†^p < 0.05 versus si-Slc5a2-treated PTs, § p < 0.05 versus Ald and si-Slc5a2-treated PTs.

We examined the association between SGLT2 and the stimulatory effects of Ald on Na^+^ reabsorption in PTs. In PTs cultured in the Dapa-supplemented culture medium, Ald-induced Na^+^ reabsorption was completely suppressed (Figures 2A,B). We then examined the effect of Ald on Na^+^ reabsorption using isolated PTs, in which SGLT2 expression was specifically suppressed with siRNA (Supplementary Figure S1B). The stimulatory effects of Ald on Na^+^ reabsorption in the PTs were completely inhibited due to this suppression (Figures 2C,D; Supplementary Figure S1B). Dapa did not affect the Ald-induced phosphorylation of SGK1 and ERK (Figure 2E; Supplementary Figure S2G,H). Ald-induced PT Na^+^ reabsorption is completely suppressed by both Dapa and SGLT2 siRNA silencing; however, Dapa did not affect the Ald-induced phosphorylation of SGK1 and ERK.

### 3.3 Ald regulates PT K^+^ channel expression via MR, and SGLT2i regulates this pathway

**FIGURE 3:**
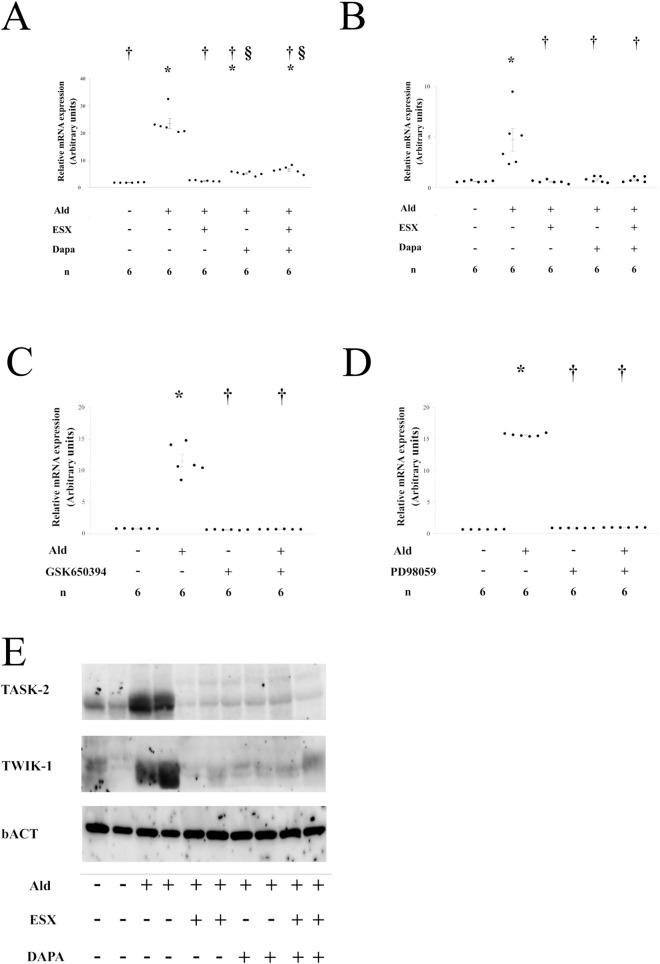
Aldosterone regulates PT K^+^ transporter expression via MR and SGLT2i regulates this pathway **(A)** Effect of esaxerenone (ESX) on aldosterone (Ald)-induced kcnk1 expression level in isolated PTs **(B)** Effect of ESX on Ald-induced kcnk5 expression level in isolated PTs, **(A,B)** *p < 0.05 versus untreated PTs; ^†^p < 0.05 versus Ald-treated PTs; § p < 0.05 versus Ald with ESX-treated PTs, **(C)** Effect of GSK650394 on Ald-induced kcnk1 expression levels in isolated PTs, **(D)** Effect of PD98059 on Ald-induced kcnk1 expression levels in isolated PTs, **(C,D)** *p < 0.05 versus untreated PTs; ^†^p < 0.05 versus Ald-treated PTs, **(E)** Effects of Ald, ESX, and Dapa on TWIK1 and TASK-2 expression in proximal tubule bundles.

The relationship between SGLT2 and Na^+^ and K^+^ transport via the Ald/MR pathway in PTs was evaluated. In isolated PTs cultured in an Ald-supplemented medium, only the mRNA expression of *Kcnk1* and *Kcnk5* was significantly upregulated compared with that in the control medium (P < 0.01) (Figures 3A,B; Supplementary Figures S3A-C). Treatment with either ESX or Dapa alone did not affect the mRNA expression of *Kcnk1* and *Kcnk5* (Supplementary Figures S3D,E). Ald-induced *Kcnk5* expression was completely suppressed in both ESX- and Dapa-supplemented medium. However, Ald-induced *Kcnk1* expression was almost completely suppressed in the presence of ESX, whereas a small but statistically significant expression persisted in the Dapa-supplemented medium (Figures 3A,B). The influence of SGK1 and ERK, previously implicated in Ald-mediated Na^+^ transport in PTs, on *Kcnk1* expression was assessed. In isolated PTs cultured with GSK650394 (an SGK1 inhibitor) or PD98059 (an ERK inhibitor), Ald-induced upregulation of *Kcnk1* mRNA expression was significantly suppressed (Figures 3C,D). In bundles of PTs, Ald increased TWIK-1 and TASK-2 mRNA and protein expression, an effect completely suppressed by ESX treatment. However, co-treatment with Dapa partially preserved TWIK-1 expression (Figure 3E; Supplementary Figures S3F,G). Ald upregulated TWIK-1 and TASK-2 expression, an effect suppressed by ESX and inhibitors of SGK1 and ERK, while co-treatment with Dapa partially preserved TWIK-1 expression.

### 3.4 ESX improves DKD-like pathology but causes hyperkalemia

SDT fatty rats subjected to unilateral nephrectomy (UNx) and continuous Ald administration developed hypertension, hyperinsulinemia, and significant renal injury, consistent with the pathological features of DKD. These abnormalities were significantly ameliorated by ESX monotherapy; however, this beneficial effect was accompanied by the development of hyperkalemia (Table 1). Given the significant difference in BW among the groups, which was a potential confounding factor, we performed an analysis of covariance (ANCOVA) on all *in vivo* parameters using BW as a covariate. The detailed statistical results of the ANCOVA are presented in Supplementary Table S1. The ANCOVA confirmed that even after statistically adjusting for the influence of BW, the main effect of Group (treatment effect) remained highly significant (P < 0.05) for key parameters, including systolic blood pressure, serum creatinine levels, kidney weight, fasting serum insulin level, and histological scores (Supplementary Table S1). Therefore, the *in vivo* parameters are presented as the LS means ± SEM, derived from the ANCOVA model (Table 2). ESX monotherapy significantly ameliorated DKD pathology but resulted in a significant elevation of serum K^+^ level compared to controls.

### 3.5 SGLT2i suppresses MRB-induced hyperkalemia

The ANCOVA revealed a highly significant difference in serum K+ level among the treatment groups (P < 0.001). Posthoc analysis showed that ESX monotherapy significantly elevated serum K+ level in Ald-treated SDT fatty rats compared with that in the control group (P < 0.001). In contrast, the Ald + ESX + Dapa therapy effectively prevented this elevation. The serum K^+^ level in the ESX + Dapa group was not significantly different from that in the control group, confirming the maintenance of normokalemia. Furthermore, a significant difference was observed between the ESX group and the ESX + Dapa group (Table 2; Supplementary Table S1). ESX monotherapy caused hyperkalemia; however, the combination of ESX + Dapa effectively prevented this elevation, which resulted in the maintenance of normokalemia.

### 3.6 Effects of ESX + Dapa therapy on renal K^+^ channels

**FIGURE 4:**
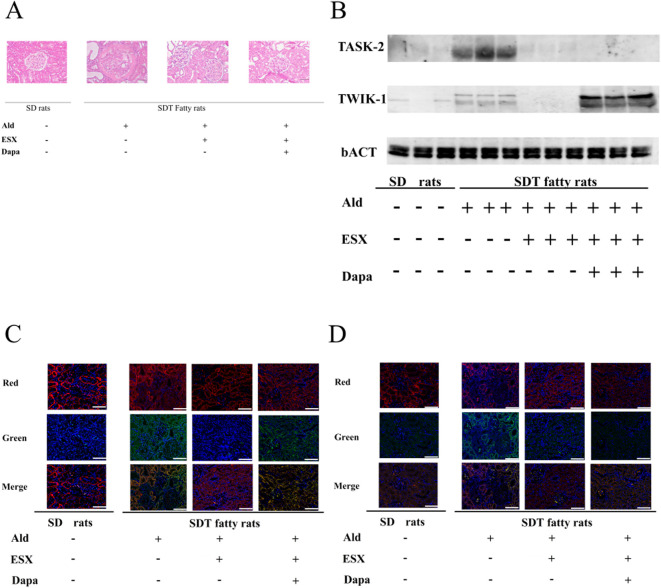
Effects of mineralocorticoid receptor inhibition *in vivo* **(A)** Pathological image of a rat kidney. Periodic acid-Schiff staining image is shown. Rats continuously treated with aldosterone showed marked mesangiolysis, polar vasculosis, and tubulointerstitial lesions, and considerable improvement in renal pathology following treatment with esaxerenone with or without dapagliflozin. Horizontal bars represent 50 µm **(B)** TWIK-1 and TASK-2 expression on kidney cortex **(C)** Immunofluorescence staining of TWIK-1 in rat renal cortex. Red in the top panel shows phalloidin staining; green in the middle panel shows TWIK-1 staining, along with nuclear staining with 4′,6-diamidino-2-phenylindole (DPAI); and the bottom panel shows the merged version. Horizontal bars represent 50 µm **(D)** Immunofluorescence staining of TASK-2 in rat renal cortex. Red in the top panel shows phalloidin staining; green in the middle panel shows TASK-2 staining, along with nuclear staining with DPAI, and the bottom panel shows the merged version. Horizontal bars represent 50 µm.

To explore the mechanisms underlying the effect of ESX + Dapa combination therapy on renal K^+^ homeostasis and potential differences from ESX monotherapy, the expression levels of renal K^+^ channels were evaluated using Western blotting and immunofluorescence analysis. The expression of TWIK-1 and TASK-2 was elevated in Ald-treated SDT fatty rats compared with that in control rats. This upregulation was completely suppressed by ESX monotherapy. In contrast, TWIK-1 expression did not significantly decrease in the ESX + Dapa group compared with that in the ESX group of Ald-treated SDT fatty rats (Figures 4B–D; Supplementary Figures S4a-d). *In vivo*, Ald increased TWIK-1 and TASK-2 expression, which ESX suppressed; notably, TWIK-1 expression in the ESX + Dapa group was significantly higher than in the ESX monotherapy group.

### 3.7 Renoprotective effect of ESX + Dapa therapy

Beyond normalizing serum K^+^ level, the ESX + Dapa therapy significantly mitigated the incidence of hyperkalemia and offered superior renoprotection compared with ESX monotherapy. Specifically, the LS means for SBP, serum creatinine levels, and glomerular sclerosis index in the ESX + Dapa group were significantly lower than those in the Ald-treated group and approached levels observed in the control group. Histopathological examination of kidneys from Ald-treated SDT fatty rats revealed pronounced glomerular and tubulointerstitial injury, which was significantly attenuated by ESX + Dapa therapy (Table 2). ESX + Dapa combination therapy resulted in superior renoprotective benefits compared with ESX monotherapy.

## 4 Discussion

### 4.1 Overview of the findings

Through the *ex vivo* examinations of isolated PTs, we confirmed the existence of the MR signaling pathway and also confirmed that Ald activates this pathway via SGK1/ERK, resulting in enhanced PT Na^+^ reabsorption. In addition, Ald regulates TWIK-1, a K^+^-excretion channel expressed in the PTs; TWIK-1 is particularly important for MR-mediated K^+^ regulation by Ald. The *in vivo* experiments revealed that MRB-induced hyperkalemia was significantly suppressed when ESX was combined with Dapa, resulting in a greater amelioration of kidney injury, compared to ESX alone. These results suggest that MRB-induced hyperkalemia involves K^+^ channels expressed in the PT, with SGLT2i potentially modulating their expression.

### 4.2 Novel discovery of the Ald/MR signaling axis and Na^+^ transport regulation in PTs

Despite the role of the Ald/MR pathway in the distal nephron and the non-MR Rac1-mediated pathway in the pathogenesis of hypertension and hypokalemia being well-established (Hirohama et al., 2021; Bazard et al., 2020; Shibata et al., 2008), the function of MR and Ald-mediated electrolyte transport in the PTs remains unclear (Salyer et al., 2013; Girard et al., 2003). To the best of our knowledge, the direct influence of the Ald/MR signaling axis on Na^+^ transporter activity in the PT has not been previously established. This knowledge gap is partially attributable to the limited MR selectivity of traditional MRBs such as spironolactone and eplerenone, which require high reagent concentrations for *ex vivo* examination. Such high concentrations frequently induce off-target pharmacological effects and solvent-associated cytotoxicity, thereby impeding the accurate assessment of Na^+^ transport in isolated PTs. ESX, utilized in this study, exhibits high MR selectivity and is effective at physiologically relevant concentrations, enabling reliable *ex vivo* analyses.

Based on the abovementioned attributes, Ald was found to activate the MR signaling pathway in isolated PTs via SGK1/ERK, culminating in the increased reabsorption of Na^+^. Studies in cultured PT cells have demonstrated Ald-mediated activation of NHE3, NBCe1, and H^+^-ATPase (Salyer et al., 2013; Leite-Dellova et al., 2011; Pergher et al., 2009; Drumm et al., 2006). Additionally, Ald has been shown to increase Na^+^/K^+^ -ATPase activity and stimulate NHE3 via SGK1, a classic MR-dependent pathway in PTs (Salyer et al., 2013). Consistent with these observation, the findings of the present study confirm Ald-induced activation of both NBCe1 and NHE3 in freshly isolated PTs. Furthermore, the SGK1/ERK1/2 signaling axis was identified as a downstream effector of Ald-mediated MR activation in PTs. Thus, our findings strongly suggest the existence of a novel Ald/MR regulatory pathway for PT Na^+^ transport. This pathway is consistent with the known regulatory mechanism of MRs in other renal segments and the documented regulation of PT transporter expression by systemic hormones, supporting the novelty of this observation. This study established a novel Ald/MR signaling pathway in the PTs, which activates NBCe1 and NHE3 via the SGK1/ERK axis to enhance Na^+^ reabsorption.

### 4.3 Critical roles of SGK1/ERK signaling in ald-induced organ damage and PT transport

SGK1 plays an important role in Ald signaling; Ald activates SGK1 and can regulate the surface expression and function of many ion channels within kidney cells (Valinsky et al., 2018; Liang et al., 2010). Moreover, SGK1 is associated with increased fibrotic mediators, such as connective-tissue-growth-factor and transforming growth factor-β (Martín-Fernández et al., 2014; Terada et al., 2008). In addition to its association with inflammatory (tumor necrosis factor-α and interleukin-1β) and NADPH oxidase pathways, SGK1 physically interacts with ERK1/2 and MEK1/2 and regulates ERK2 activation through phosphorylation (Hallows et al., 2010; Won et al., 2009). Furthermore, SGK1 exerts cardiac effects; Ald increases intracellular calcium levels via SGK1 and promotes the expression of pro-inflammatory and pro-fibrotic mediators in cardiomyocytes (Martín-Fernández et al., 2014). This may induce cardiac changes, such as myocardial hypertrophy, and increase the risk of heart failure and malignant arrhythmias. In unilaterally nephrectomized rats, SGK1 expression in adipose tissue correlated with serum Ald concentrations, whereas spironolactone reduces SGK1 expression and insulin signaling (Terada et al., 2008). Similarly, the rat model used in this study exhibited insulin resistance, significant urinary protein excretion, renal damage, and hypertension, all of which were ameliorated by ESX treatment. Similar to humans, ESX-treated rats in this study developed hyperkalemia; however, the incidence of hyperkalemia was significantly reduced with ESX + Dapa therapy. These findings underscore the critical role of SGK1 in Ald signaling and its contribution to Ald-induced organ damage.

ERK1/2 plays a crucial role in ion transport in PT, and we have previously demonstrated its involvement in enhancing Na^+^ reabsorption via angiotensin II and pioglitazone, a PPARγ agonist (Mizuno et al., 2022; Endo et al., 2011). Additionally, fibroblast growth factor 23 is regulated by the ERK1/2/SGK1 pathway in Na^+^-K^+^ transport within the PT (Andrukhova et al., 2012). These findings support the role of MR-mediated Ald signaling in PT Na^+^ transport and suggest the involvement of the ERK/SGK1 pathway in downstream signaling. The SGK1/ERK pathway, identified as a downstream effector of MR activation in the PTs, is consistent with known regulatory mechanisms of PT ion transport and is critical for Ald-mediated renal and systemic damage.

### 4.4 Role of PT K^+^ channels in MRB-induced hyperkalemia

The effect of Ald on the mRNA expression of K^+^ channels in the PTs was also examined. Several K^+^ channels, including KCNQ1/KCNE1, KCNA10, Kir6.1, Kir7.1, Maxi K channel, and TREK-2b/TASK, are expressed in the PT, though their regulatory mechanisms and physiological significance remain poorly understood (Hebert et al., 2005). Among these channels, TWIK-1/*Kcnk1* and TASK-2/*Kcnk5* were prioritized for investigation. TWIK-1/*Kcnk1* is expressed in the PTs, thin and thick ascending limbs, distal tubules, and medullary collecting ducts, playing a critical role in MR-mediated K^+^ regulation by Ald. Although TWIK-1 is not exclusively expressed in the PT, in mice, the knockout of TWIK-1/*Kcnk1* results in hypophosphatemia owing to impaired phosphate transport in the PT (Nie et al., 2005), suggesting the role of TWIK-1 in the membrane trafficking of transport molecules in PTs and its potential link with SGLT2. Conversely, TASK-2/*Kcnk5* knockout mice exhibit mild proximal tubular acidosis without changes in serum K^+^ levels (Warth et al., 2004). Ald was found to upregulate the expression of PT K+ channels, TWIK-1 and TASK-2, which was completely suppressed by the MRB, ESX. This finding provides a potential molecular basis for MRB-induced hyperkalemia.

### 4.5 Synergistic effect of SGLT2i in modulating K^+^ channel expression and mitigating hyperkalemia

In this study, Ald increased the expression levels of TWIK-1/*Kcnk1* and TASK-2/*Kcnk5*, whereas ESX completely suppressed their expression. This suppression of K^+^ channel mRNA expression may be associated with the development of hyperkalemia through reduced urinary K^+^ excretion. Conversely, when Dapa was administered alone or in combination with ESX, the Ald-induced expression of TWIK-1/*Kcnk1* was partially preserved. These findings suggest a potential mechanism by which SGLT2 inhibitors may alleviate hyperkalemia. In *in vivo* experiments, MRB-induced hyperkalemia was significantly attenuated when combined with Dapa, and the amelioration of renal injury was greater than that observed with ESX monotherapy. These results suggest that MRB-induced hyperkalemia involves K^+^ channels expressed in the PT and that SGLT2is may modulate their expression. Crucially, the addition of Dapa partially preserved TWIK-1 expression, and *in vivo*, this combination significantly attenuated ESX-induced hyperkalemia, highlighting a synergistic mechanism where SGLT2i modulates PT K^+^ transport.

### 4.6 Hypothesized dual-mechanism for SGLT2i-mediated hyperkalemia mitigation and non-MR regulation of TWIK-1

While our findings suggest that SGLT2 inhibition may directly modulate the expression of K^+^ channels in the PTs, other alternative or complementary mechanisms may be involved. Conversely, when Dapa was administered alone or together with ESX, the Ald-induced expression of TWIK-1/Kcnk1 was partially preserved. These findings suggest a potential mechanism by which SGLT2is may alleviate hyperkalemia. To clarify how Dapa mitigates the incidence of ESX-induced hyperkalemia, we propose a dual mechanism-of-action involving both proximal and distal renal segments. First, the PT mechanism: our *ex vivo* data strongly suggest a novel local effect where Dapa partially prevents the ESX-induced suppression of TWIK-1 expression. By modulating the local K^+^ channel expression in the PTs, Dapa may directly counteract the K^+^-retention tendency caused by MRBs in this segment. The second is the distal nephron (indirect) mechanism: SGLT2 inhibition reduces Na^+^ reabsorption in the PTs, thereby enhancing the delivery of Na^+^ and fluid to the downstream regions of the distal convoluted tubules and collecting ducts. This increased Na^+^ load promotes Na^+^ reabsorption (epithelial Na^+^ channel) in exchange for K^+^ secretion (via renal outer medullary K^+^ channel). Given that the aldosterone-sensitive-distal nephrons are the primary site of K^+^ secretion, this well-established indirect mechanism likely plays a dominant role in promoting kaliuresis and alleviating hyperkalemia under MRB therapy (Palmer and Clegg, 2024; Neuen et al., 2022, Ishigami et al., 2020). Therefore, the observed amelioration of hyperkalemia is likely a synergistic effect of a novel PT transporter modulation (direct or local effect) and the enhanced distal Na^+^ delivery (indirect, systemic effect). This dual mechanism-of-action is supported by our *in vivo* analysis data, which show that ESX + Dapa therapy maintains normokalemia (Table 2).

Furthermore, the findings suggest that the Ald-induced upregulation of *Kcnk1* may not be mediated through the MRs. For instance, under acidic conditions or low extracellular K^+^ concentrations, TWIK-1/*Kcnk1* becomes permeable to sodium and is activated by protein kinase C (PKC) (Lesage et al., 1996; Chen et al., 2014). However, Ald did not alter the pHi, although previous studies reported that Ald influences pHi and activates PKC (Winter et al., 2011). Therefore, PKC activation by Ald may play a role in the regulation of TWIK-1/*Kcnk1* in the PTs. Additionally, Rac1 has been implicated in this process, as a recent study reported that Rac1 is involved in the endocytosis-mediated regulation of kir2.1 expression (Hager et al., 2022). Dapa likely mitigates ESX-induced hyperkalemia through a synergistic dual mechanism: a novel local effect involving PT K^+^ channel modulation, which is a direct effect, and the well-established systemic effect of enhanced distal Na^+^ and fluid delivery, which is an indirect effect.

### 4.7 Limitations and future directions

This study had some limitations. First, the localization of MR receptors in PTs could not be demonstrated pathologically owing to the unavailability of high-quality MR antibodies. However, we were able to measure and evaluate mRNA expression levels by manual isolation. Second, K^+^ channels were evaluated based on mRNA and protein expression levels, a common approach in studies focusing on their pathophysiological expression and function correlation, although K^+^ channel activity was not directly measured (Nie et al., 2005; Li et al., 2019; Zou et al., 2022; Zhang et al., 2024) Third, although our *ex vivo* data strongly suggest that the Ald/MR signaling axis modulates the expression of PT Na^+^ and K^+^ transporters, it is difficult to precisely evaluate the extent to which these PT transporters contribute to the overall systemic electrolyte balance and K^+^ excretion shown in Tables 1 and 2. K^+^ metabolism in a living organism is a complex process involving multiple organs and transporters, particularly the distal nephron, which is the primary site of K^+^ secretion. Determining the precise physiological contribution of PT transporters to systemic K^+^ excretion would require specific PT-targeted knockout animal models. Therefore, the exact *in vivo* contribution remains a subject for future studies. Finally, we primarily focused on investigating the synergistic effects of Esx and Dapa, particularly their ability to alleviate the hyperkalemia associated with MRB monotherapy. Therefore, we did not include a group treated with Ald + Dapa. While including such a group would provide valuable insights into the direct effects of Dapa on Ald-induced ion transport, its absence does not undermine our primary conclusion that the combined therapy effectively prevents the hyperkalemic side effect of Esx. Our findings strongly support the use of Esx and Dapa together to enhance the safety and efficacy of MRB therapy for conditions such as DKD. Future studies should explore the direct effects of SGLT2is on renal ion transport in Ald-excess states to further clarify the mechanisms observed in our study. Despite limitations regarding direct MR localization and K^+^ channel activity measurements, this study strongly supports the combined use of ESX and Dapa; future studies should focus on PT-specific knockout models and the direct effects of SGLT2is on Ald-induced transport to fully clarify the *in vivo* contributions.

### 4.8 Conclusion

In summary, this study demonstrated that MR is expressed at the mRNA level in the PTs, where Ald promotes Na^+^ reabsorption through NBCe1 and NHE3 and regulates K^+^ channel expression via the ERK/SGK1 signaling axis. Ald primarily regulates PT K^+^ channel expression, particularly TWIK-1 expression, through MR activation, thereby significantly influencing K^+^ excretion within the PT. The *ex vivo* findings were supported via the *in vivo* experiment findings, demonstrating that Dapa effectively attenuates ESX-induced hyperkalemia and ameliorates renal injury. These results highlight the role of MRBs in the pathophysiology of hyperkalemia, as mediated through PT K^+^ channels, and suggest that SGLT2is may serve as potential modulators of PT K^+^ transport. Furthermore, the combination of ESX and Dapa confers superior renoprotective effects than ESX monotherapy and may mitigate the incidence of MRB-induced hyperkalemia when co-administered with an SGLT2i. However, further large-scale clinical trials will be necessary to validate these findings.

## Data Availability

The original contributions presented in the study are included in the article/Supplementary Material, further inquiries can be directed to the corresponding author.
