# Supplementary material for: Combined effect of esaxerenone and dapagliflozin on aldosterone-mediated sodium reabsorption and potassium excretion
Source: Front Physiol. 2025 Dec 11;16:1677518. doi: 10.3389/fphys.2025.1677518 (PMC12738813; doi:10.3389/fphys.2025.1677518)
Supplement: Supplementary file 1 [file DataSheet1.docx]

**Supplementary Material**

**Supplementary Methods**

**Supplementary Figure1.** Effect of small interfering RNA in isolated rat proximal tubules

**Supplementary Figure2.** Effect of aldosterone specific inhibitors on aldosterone-induced SGK1 and ERK phosphorylation

**Supplementary Figure3.** Effect of aldosterone on K-channel expression in proximal tubules

**Supplementary Figure4.** Effect of aldosterone on K-channel expression in proximal tubules

**Supplementary Table 2.**

**Supplementary Reference**

**Supplementary Methods**

**Western blotting**

Thin slices of kidney cortex were excised from rats and divided into small bundles following the method outlined by Nakamura M et al. (2015, 2020) and Mizuno et al. (2022). The bundles were homogenized in ice-cold buffer A [25 mM Tris-HCl (pH 7.4), 10 mM sodium orthovanadate, 10 mM sodium pyrophosphate, 100 mM sodium fluoride, 10 mM ethylenediaminetetraacetic acid (EDTA), 10 mM ethylene glycol-bis(2-aminoethylether)-N,N,N',N'-tetraacetic acid (EGTA), and 1 mM phenylmethylsulfonyl fluoride], followed by centrifugation at 12,000 × *g* for 10 min at 4 °C. The supernatants were collected for subsequent analysis.

Samples were incubated in DMEM at 37 °C under 5% CO_2_ for 40 min with inhibitors, such as 3 µM ESX, 10 µM SGK650394 (FUJIFILM Wako Pure Chemical Corp., Osaka, Japan), 10 µM PD98059 (FUJIFILM Wako Pure Chemical Corp., Osaka, Japan), and 50 µM Dapa, or without the inhibitors and for 15 min in DMEM containing 1 nM aldosterone (Ald). Following incubation, the samples were homogenized in ice-cold buffer A (25 mM Tris-HCl [pH 7.4], 10 mM sodium orthovanadate, 10 mM sodium pyrophosphate, 100 mM sodium fluoride, 10 mM EDTA, 10 mM EGTA, and 1 mM phenylmethylsulfonyl fluoride) and centrifuged at 12,000 × *g* for 10 min. The supernatants from each sample were collected and divided into aliquots containing equal amounts (approximately 20 µg) of protein. The samples were separated using 10% sodium dodecyl sulfate-polyacrylamide gel electrophoresis and transferred to nitrocellulose membranes. Membranes were blocked with 5% skim milk in Tris-buffered saline (137 mM NaCl, 2.68 mM KCl, 25 mM Tris, adjusted to pH 7.4), incubated overnight at 4 °C with primary antibodies, and incubated with horseradish peroxidase (HRP)-conjugated secondary antibodies at 25℃ for 1 h. Primary antibodies against SGK1 (#12103), phospho-SGK1 (Thr256) (#29239), ERK1/2 (#9102), phospho-ERK1/2 (Thr202/Tyr204) (#9101), beta-actin (#4970) were purchased from Cell Signaling Technology (Danvers, MA, USA). Antibodies against TWIK-1 (sc-517040) and TASK-2 (SC-271836) were purchased from Santa Cruz Biotechnology (Santa Cruz, CA, USA). The HRP-conjugated anti-rabbit IgG antibody (111-035-003) was purchased from Jackson ImmunoResearch Laboratories (West Grove, PA, USA). Protein bands were detected using a chemiluminescence detection system (ImageQuant LAS 4000 Mini; GE Healthcare, Little Chalfont, UK). For quantification of protein expression all western blotting data were normalized against their respective internal loading controls (β-actin) to account for variations in protein loading. Because all samples could not be scanned on a single gel, quantitative data were obtained from multiple blots. Normalization against the internal control within each gel enabled reliable comparisons of protein levels across different gels. Final quantitative data are presented as means ± the standard error of the mean (SEM). Although western blotting confirms the expression level of the K^+^ channel protein, this analysis does not provide a direct measure of its functional activity or ion channel capability. Further functional assays would be required to fully assess the physiological activity.

**Immunohistochemistry studies**

For immunohistochemical analysis of transporter expression, kidneys from model rats were fixed in 4% buffered paraformaldehyde, paraffin-embedded, and sectioned at 5 µm thickness. Sections were air-dried at 25 °C, rehydrated in phosphate-buffered saline (PBS), and incubated overnight at 4 °C with primary antibodies against anti-TWIK-1 (sc-517040, Santa Cruz Biotechnology, CA, USA) and anti-TASK-2 (sc-s71836, Santa Cruz Biotechnology, CA, USA). The sections were then incubated with a mixture of Alexa Fluor 488 anti-rabbit or anti-goat IgG, Alexa Fluor 568 phalloidin for F-actin labeling, and 4',6-diamidino-2-phenylindole (DAPI) for nuclear labeling for 60 min at 27 °C. The samples were observed and evaluated under a confocal laser scanning microscope (Olympus, Tokyo, Japan).

**Quantitative pathological analysis**

***Glomerular sclerosis index***

Glomerular pathology was assessed using the glomerular sclerosis index following the method outlined by Raij et al(Raij et al., 1984). The scoring criteria were as follows: no change (–), mild mesangial expansion (+), segmental sclerosis (++), and global sclerosis (+++). Fifty glomeruli were selected from the renal cortex of each rat, and each was graded on a scale of 1 to 4 according to the severity of glomerular sclerosis. The mean score per rat was calculated and reported as the glomerular sclerosis index.

***Tubular injury score***

PAS-stained samples were visualized using a digital microscope camera at 100× magnification. Ten arbitrary non-overlapping areas were imaged. The percentage of the area occupied by tubular injury was semi-quantified and calculated as a tubular injury score as follows: 0; no tubular injury, 0.5; < 5%, 1.0; 5–20%, 1.5; 21–35%, 2.0; 36–50%, 2.5; 51–65%, 3.0; > 65% (Raij et al., 1984).

**Blood pressure measurement**

Systolic blood pressure was measured in conscious rats using the tail-cuff method (BP-98A, Softron Co., Ltd.). Measurements were taken at the beginning of the treatment period, and every 2 weeks thereafter. The average of three stable measurements was recorded for each rat.

**Biochemical parameter measurements**

Fasting blood samples were collected from the tail vein after a 12-hour fast. Plasma insulin concentrations were measured using an enzyme-linked immunosorbent assay (ELISA) kit (Shibayagi Co., Ltd., Gunma, Japan) in accordance with the manufacturer’s instructions. At the time of euthanasia, blood samples were collected from the vena cava after anesthesia. Plasma levels of blood urea nitrogen (BUN), creatinine, potassium, and glucose were immediately measured using the i-STAT^®^ handheld clinical analyzer with the CHEM8+ cartridge (Abbott Laboratories, Abbott Park, IL, USA).

**Randomization in the *in vivo* experiments**

In the context of the *in vivo* experiments, randomization was conducted as follows: Forty-four rats were divided into four groups. Additionally, all SDT fatty rats were assigned a random number generated using the standard = RAND() function in Microsoft Excel; the rats were then assigned a cage number based on their position on the rack.

**Supplementary Figure1.** Effect of small interfering RNA in isolated rat proximal tubules

**
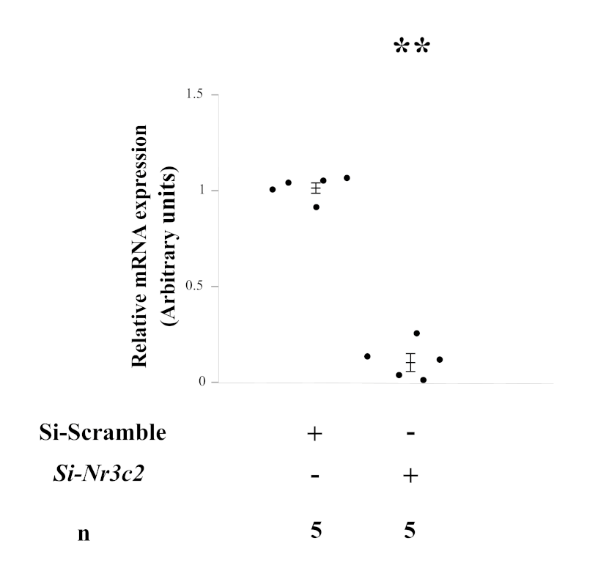
A**

**B**

**
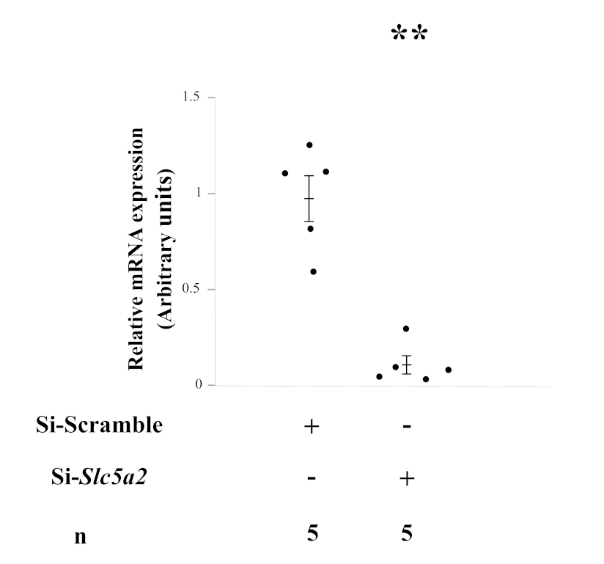
**

**Supplementary Figure1.** Effect of small interfering RNA in isolated rat proximal tubules

1. Effect of small interfering RNA against *Nr3c2* in the isolated rat PTs
2. Effect of small interfering RNA against *Slc5a2* in the isolated rat PTs

** p < 0.01 versus control PTs treated with scrambled siRNA.

**Supplementary Figure2. Effect of Aldosterone specific inhibitors on aldosterone-induced SGK1 and ERK phosphorylation**

**A**

**
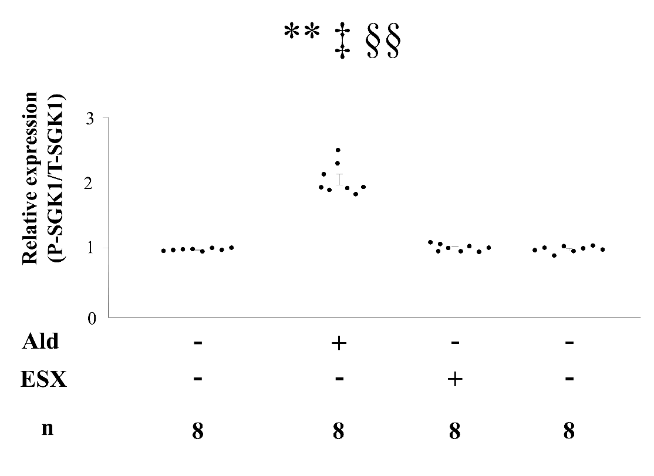
**

**
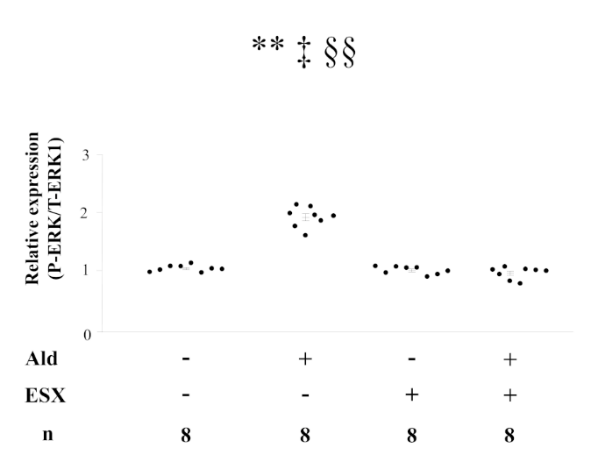
B**

**C**

**
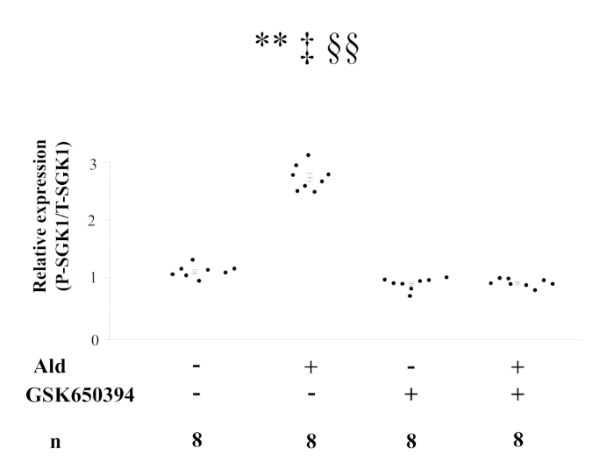
**

**
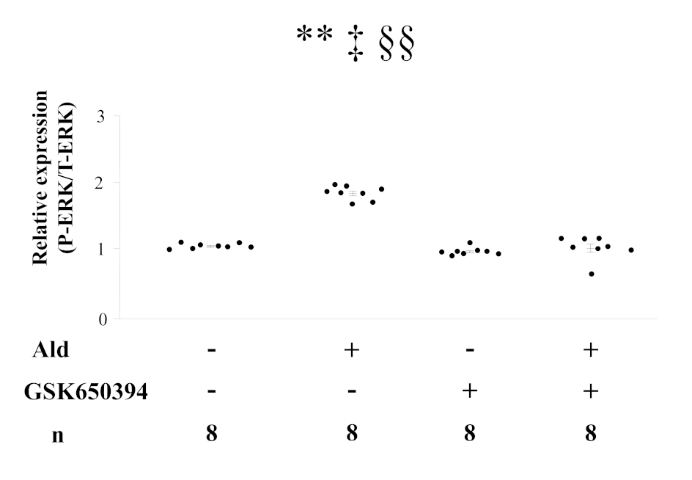
D**

**E**

**
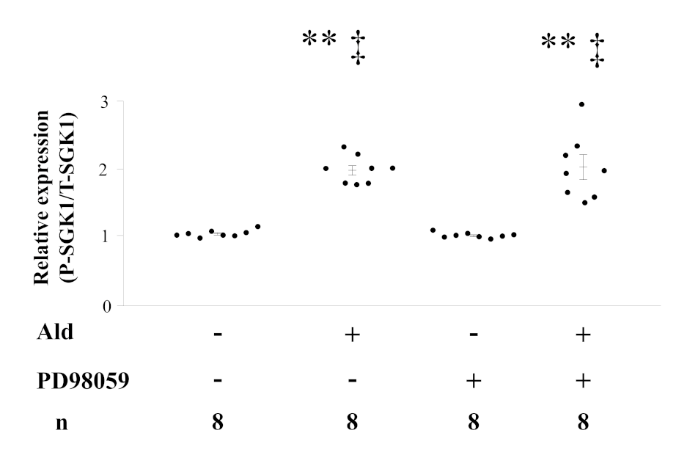
**

**F**

**
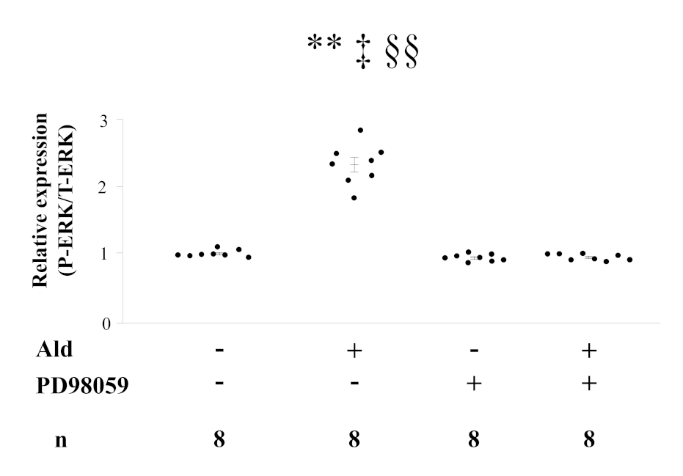
**

**G**

**
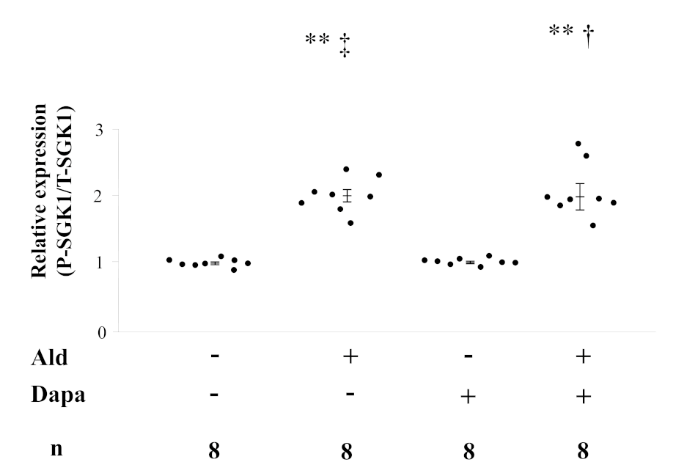
**

**H**

**
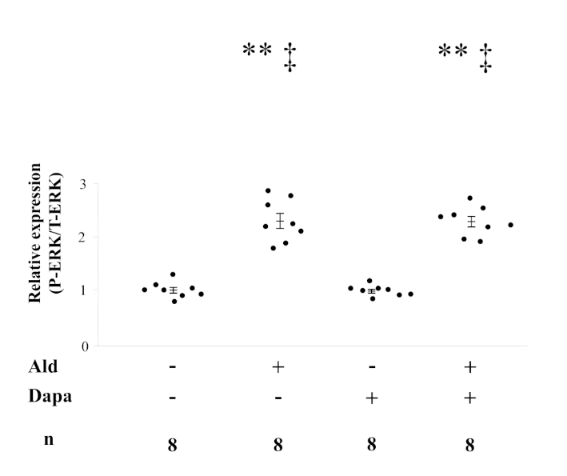
**

**Supplementary Figure2. Effect of aldosterone-specific inhibitors on aldosterone-induced SGK1 and ERK phosphorylation**

1. Effect of esaxerenone (ESX) on aldosterone (Ald)-induced SGK1 phosphorylation in rat renal cortex tissue (n = 8). **p < 0.01 versus untreated renal cortex tissue; ^‡^p < 0.01 versus ESX-treated renal cortex tissue; ^§§^p < 0.01 versus Ald and ESX-treated renal cortex tissue.
2. Effect of ESX on Ald-induced ERK phosphorylation in rat renal cortex tissue (n = 8). **p < 0.01 versus untreated renal cortex tissue; ^‡^p < 0.01 versus ESX-treated renal cortex tissue; ^§§^p < 0.01 versus Ald and ESX-treated renal cortex tissue.
3. Effect of GSK650394 on Ald-induced SGK1 phosphorylation in rat renal cortex tissue (n = 8). **p < 0.01 versus untreated renal cortex tissue; ^‡^p < 0.01 versus GSK650394-treated renal cortex tissue; ^§§^p < 0.01 versus Ald and GSK650394-treated renal cortex tissue.
4. Effect of GSK650394 on Ald-induced ERK phosphorylation in rat renal cortex tissue (n = 8). **p < 0.01 versus untreated renal cortex tissue; ^‡^p < 0.01 versus GSK650394-treated renal cortex tissue; ^§§^p < 0.01 versus Ald and GSK650394-treated renal cortex tissue.
5. Effect of PD98059 on Ald-induced SGK1 phosphorylation in rat renal cortex tissue (n = 8). **p < 0.01 versus untreated renal cortex tissue; ^‡^p < 0.01 versus PD98059-treated renal cortex tissue.
6. Effect of PD98059 on Ald-induced ERK phosphorylation in rat renal cortex tissue (n = 8). **p < 0.01 versus untreated renal cortex tissue; ^‡^p < 0.01 versus PD98059-treated renal cortex tissue; ^§§^p < 0.01 versus Ald and PD98059-treated renal cortex tissue.
7. Effect of dapagliflozin (Dapa) on Ald-induced SGK1 phosphorylation in rat renal cortex tissue (n = 8). **p < 0.01 versus untreated renal cortex tissue; ^†^p < 0.05 versus Dapa-treated renal cortex tissue; ^‡^p < 0.01 versus Dapa-treated renal cortex tissue.
8. Effect of Dapa on Ald-induced ERK phosphorylation in rat renal cortex tissue (n = 8). **p < 0.01 versus untreated renal cortex tissue; ^‡^p < 0.01 versus Dapa-treated renal cortex tissue.

**Supplementary Figure3. Effect of aldosterone on K-channel expression in proximal tubules**

**
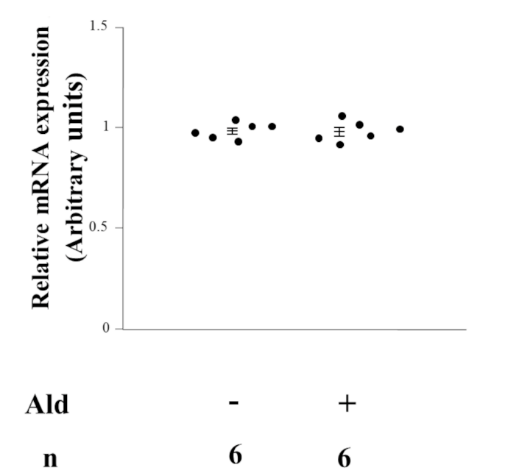
A**

**
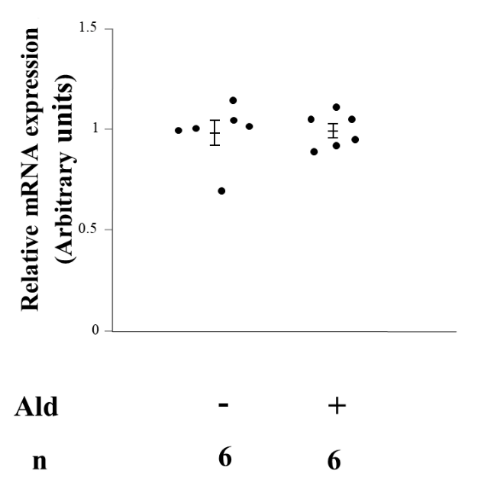
B**

**C**


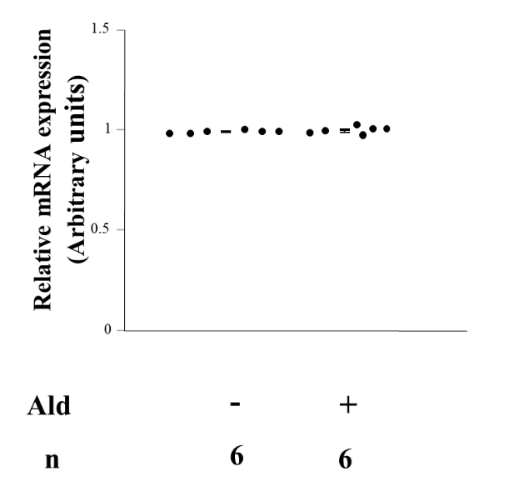


**D**


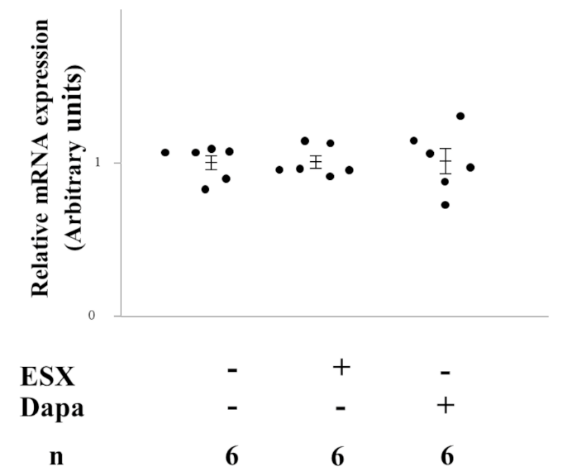


**E**


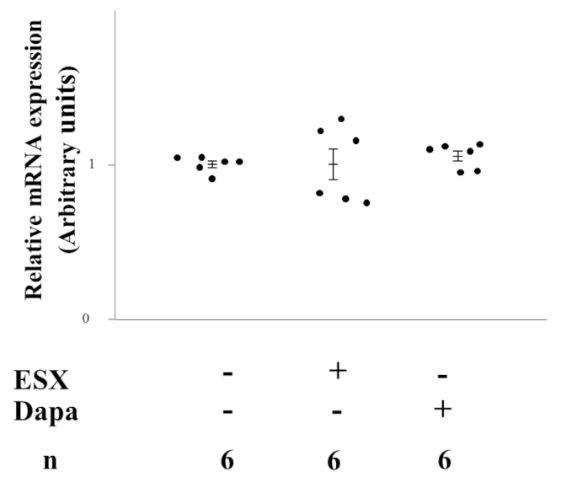


**F**


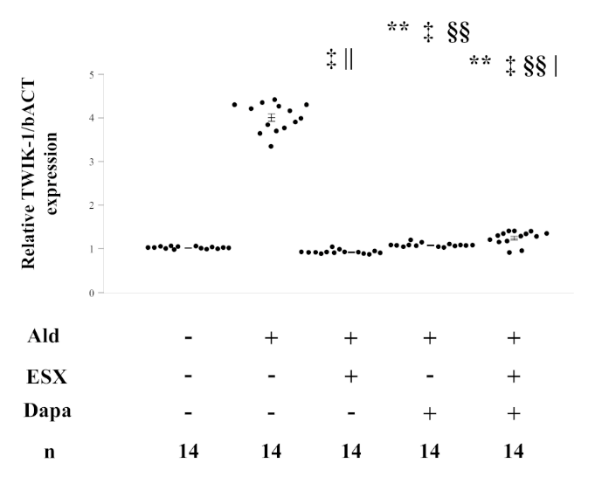


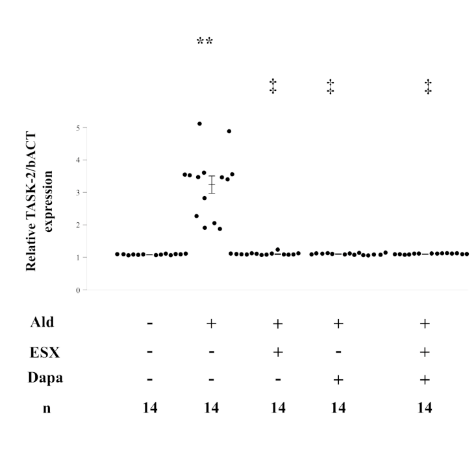
**G**

**Supplementary Figure3. Effect of aldosterone on K-channel expression in proximal tubules**

1. Effect of aldosterone (Ald) on *kcnq1* mRNA expression in proximal tubules (PTs)
2. Effect of Ald on *kcnma1* mRNA expression in the PTs
3. Effect of Ald on *kcne3* mRNA expression in the PTs
4. Effect of esaxerenone (ESX) or dapagliflozin (Dapa) on *kcnk1* mRNA expression in the PTs
5. Effect of ESX or Dapa on *kcnk5* mRNA expression in the PTs
6. Effect of ESX or Dapa on TWIK-1 expression in the PTs
7. Effect of ESX or Dapa on TASK-2 expression in the PTs

*p < 0.05 versus untreated PTs; **p < 0.01 versus untreated PTs; ^†^p < 0.05 versus Ald-treated PTs; ^‡^p < 0.01 versus Ald-treated PTs; ^§^p < 0.05 versus Ald and ESX-treated PTs; ^§§^p < 0.01 versus Ald and ESX-treated PTs; ^|^ p <0.05 versus Ald and Dapa-treated PTs; ^||^ p <0.01 versus Ald and Dapa-treated PTs.

(F) Western blot analysis of TWIK-1 protein expression in rat PTs. The graph shows relative TWIK-1 protein levels normalized to β-actin. Data are presented as means ± the SEM, and *n* = 14 per group (total *n* = 70). The data were obtained from multiple blots, and samples were normalized to a loading control within each gel to allow for cross-blot comparisons.

(G) Western blot analysis of TASK-2 protein expression in rat kidney. The bar graph shows relative TASK-2 protein levels normalized to β-actin. Data are presented as means ± the SEM, and *n* = 14 per group (total *n* = 70). Data were obtained from multiple blots, and samples were normalized to a loading control within each gel to allow for cross-blot comparisons.

**Supplementary Figure4. Effect of aldosterone on K-channel expression in proximal tubules**


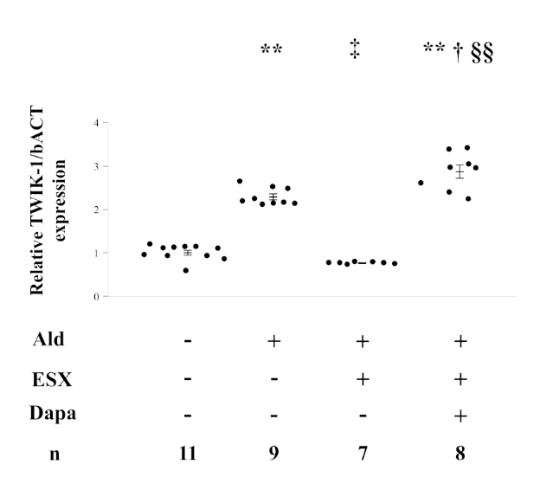


**A**

**B**


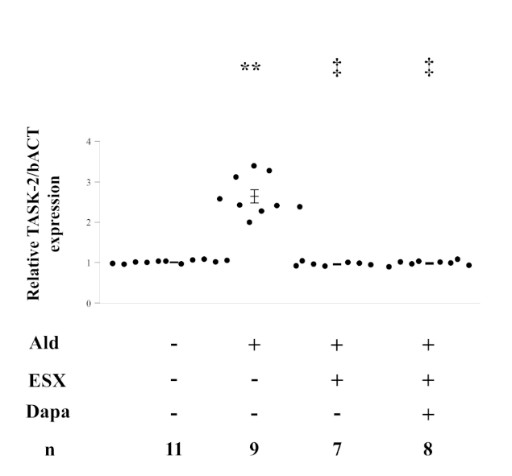


**C**


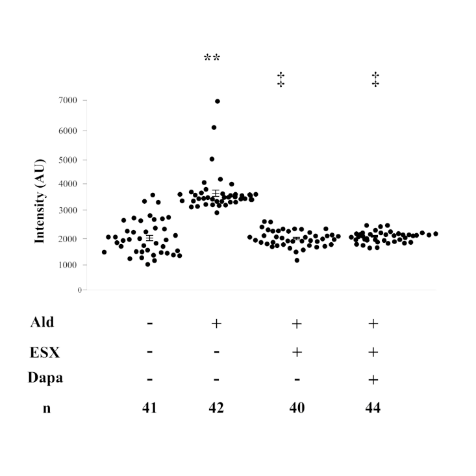


**D**


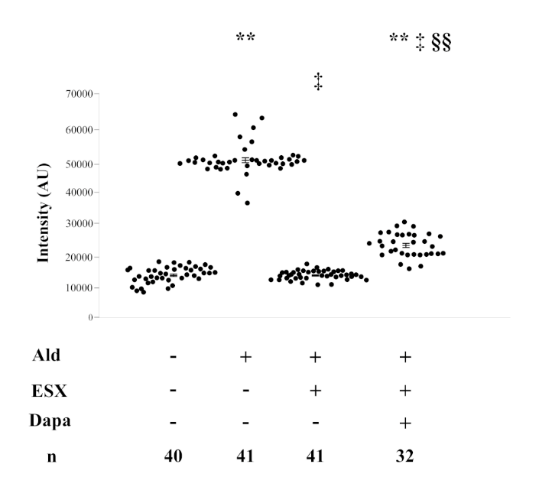


**Supplementary Figure4. Effect of aldosterone on K-channel expression in proximal tubules**

1. Protein expression in the renal cortex in *in vivo* experiments; the effect of aldosterone (Ald), esaxerenone (ESX) and dapagliflozin (Dapa) on TWIK-1 expression in the rat renal cortex (*n* = 11–7).
2. Protein expression in the renal cortex in *in vivo* experiments; the effect of Ald, ESX and Dapa on TASK-2 expression in the rat renal cortex (*n* = 11–7).
3. Results of the TWIK-1 fluorescence intensity quantification.
4. Results of the TASK-2 fluorescence intensity quantification.

a,b); **p < 0.01 versus SD rats; ^†^p < 0.05 versus SDT fatty rats with Ald; ^‡^p < 0.01 versus SDT fatty rats with Ald; ^§§^ p < 0.01 versus SDT fatty rats with Ald treated with ESX.

**C, D**); In total, 8–10 proximal tubules per rat were arbitrarily selected from the rat renal cortex, and the fluorescence intensity per square micrometer of proximal tubule cells was measured using ImageJ (NIH, Bethesda, MD, USA) and analyzed as arbitrary units. **p < 0.01 versus SD rats; ^†^p < 0.05 versus SDT fatty rats with Ald; ^‡^p < 0.01 versus SDT fatty rats with Ald; ^§^p < 0.05 versus SDT fatty rats with Ald and ESX; ^§§^p < 0.01 versus SDT fatty rats with Ald and ESX.

**Supplementary Table 1.** Statistical results of analysis of covariance for biological parameters, using body weight as a covariate

| **Parameter** | **DF** | **F-Ratio** | **P-value**  **Group×BW Interaction** | **P-value**  **Group main effect** | **P-value**  **BW main effect** | **ANCOVA**  **status** |
| --- | --- | --- | --- | --- | --- | --- |
| **sBP (mmHg)** | 3, 27 | 40.29 | 0.514 | <0.001 | 0.034 | Applied |
| **dBP (mmHg)** | 3, 27 | 11.30 | 0.293 | <0.001 | 0.699 | Applied |
| **BUN (mg/dL)** | 3, 27 | 0.837 | 0.0750 | 0.0033 | 0.998 | Applied |
| **Cre (mg/dL)** | 3, 27 | 14.32 | 0.634 | <0.001 | 0.0362 | Applied |
| **K (mmol/L)** | 3, 27 | 0.97 | 0.084 | <0.001 | 0.0131 | Applied |
| **HCO_3_^-^ (mmol/L)** | 3, 27 | 4.03 | 0.904 | <0.001 | 0.683 | Applied |
| **Fasting BS (mg/dL)** | 3, 27 | 10.52 | 0.109 | <0.001 | 0.442 | Applied |
| **Fasting serum insulin (µg/mL)** | 3, 27 | 14.96 | 0.924 | <0.001 | 0.7935 | Applied |
| **Heart weight**  **(g/gBW)** | 3, 27 | 74.15 | <0.001 | N/A | N/A | I.S |
| **Kidney weight**  **(g/gBW)** | 3, 27 | 15.68 | 0.397 | <0.001 | 0.0279 | Applied |
| **FENa (%)** | 3, 27 | 1.84 | 0.281 | <0.001 | 0.0619 | Applied |
| **Urine Alb/gCre (mg/gCre)** | 3, 27 | 2.79 | 0.992 | <0.001 | 0.8731 | Applied |
| **CCr**  **(mL/min/100 g BW)** | 3, 27 | 3.60 | 0.734 | 0.026 | 0.741 | Applied |
| **Glomerular sclerosis index** | 4, 1741 | 146.54 | 0.837 | <0.001 | 0.244 | Applied |
| **Tubular injury score** | 7, 342 | 39.65 | 0.842 | <0.001 | 0.747 | Applied |

Dapa, dapagliflozin; DF, degrees of freedom; I.S, interaction significant; N/A, not applicable; ACR, albumin-to-creatinine ratio; BUN, blood urea nitrogen; BW, body weight; Cre, creatinine; sBP, systolic blood pressure; dBP, diastolic blood pressure; UV, urinary volume; BS, blood sugar.

**Supplementary Reference**

Nakamura, M., Yamazaki, O., Shirai, A., Horita, S., Satoh, N., Suzuki, M., et al. (2015). Preserved Na/HCO_3_ cotransporter sensitivity to insulin may promote hypertension in metabolic syndrome. Kidney Int. 87, 535-542. doi: 10.1038/ki.2014.351.

Nakamura, M., Tsukada, H., Seki, G., Nobuhiko, S., Mizuno, T., Fujii, W., et al. (2020). Insulin promotes sodium transport but suppresses gluconeogenesis via distinct cellular pathways in human and rat renal proximal tubules. Kidney Int. 97, 316-326. doi: 10.1016/j.kint.2019.08.021.

Mizuno, T., Satoh, N., Horita, S., Tsukada, H., Takagi, M., Sato, Y., et al. (2022). Oxidized alkyl phospholipids stimulate sodium transport in proximal tubules via a nongenomic PPARγ-dependent pathway. J. Biol. Chem. 298, 101681. doi: 10.1016/j.jbc.2022.101681

Raij, L., Azar, S., and Keane, W. (1984). Mesangial immune injury, hypertension, and progressive glomerular damage in Dahl rats. Kidney Int. 26, 137-143. doi:10.1038/ki.1984.147
